# Unleashing the Cytokine Storm: A Case of Macrophage Activation Syndrome in Systemic Lupus Erythematosus

**DOI:** 10.7759/cureus.63167

**Published:** 2024-06-25

**Authors:** Natalie Shaykh, Oshin Rai, Abhinav Karan, Khushman K Bhullar, Vanshika Tripathi, Pramod Reddy

**Affiliations:** 1 Internal Medicine, University of Florida College of Medicine – Jacksonville, Jacksonville, USA; 2 Internal Medicine, Sri Guru Ram Das Institute of Medical Sciences and Research, Amritsar, IND

**Keywords:** hemophagocytic lymphohistiocytosis (hlh), steroid induced psychosis, cytokine storming, systemic lupus erythematosus with macrophage activation syndrome, macrophage activation syndrome (mas)

## Abstract

Macrophage activation syndrome (MAS), synonymous with secondary hemophagocytic lymphohistiocytosis (HLH), is a rare and critical complication of rheumatologic disease stemming from the unregulated activation and rapid multiplication of macrophages and T lymphocytes. While it primarily manifests in children diagnosed with systemic juvenile idiopathic arthritis (sJIA), it can arise less frequently in other rheumatologic conditions. Here, we outline the clinical course, treatment, and outcome of MAS diagnosed in an 18-year-old female previously diagnosed with SLE who exhibited a unique clinical presentation.

## Introduction

Macrophage activation syndrome (MAS) is a rare, life-threatening rheumatologic complication typically associated with adult-onset Still disease (AoSD) and systemic juvenile idiopathic arthritis (sJIA) and less frequently seen in systemic lupus erythematosus (SLE) or Kawasaki disease [1]. Although infection is the most common trigger, other potential triggers for MAS include certain medications and disease flares. Here, we present the case of an 18-year-old female with a history of SLE, who developed MAS requiring emergent therapy. In MAS, the diagnosis can be complicated by the systemic clinical presentation. Our patient exhibited neuropsychiatric symptoms, prompting consideration of various differential diagnoses including prednisone-induced psychosis, lupus cerebritis, and MAS. Distinguishing lupus cerebritis from MAS can be challenging, but our diagnosis was aided by laboratory results showing improved anti-double stranded DNA levels, along with negative findings from infectious workup, lumbar puncture, brain imaging, and electroencephalogram (EEG).

## Case presentation

An 18-year-old female with a past medical history of SLE, complicated by pancytopenia and biopsy-proven class II and III lupus nephritis on chronic steroid therapy, was evaluated for acute encephalopathy. The patient had been in her usual state of health until approximately eight days before her initial presentation when she began experiencing auditory hallucinations, paranoid delusions, insomnia, and erratic behavior. She was subsequently admitted to an external hospital with presumed prednisone-induced psychosis. Despite discontinuing prednisone for several days, her delusions and hallucinations persisted. She subsequently developed fevers with worsening pancytopenia, and an absolute neutrophil count (ANC) of 700 microliters (µL), prompting the initiation of broad-spectrum antibiotics. The patient underwent an extensive evaluation of her encephalopathy at this external facility, comprising a negative EEG, computerized tomography (CT) scan of the head, magnetic resonance imaging (MRI) of the brain, and urine drug screen along with an unremarkable infectious workup, including blood cultures, human immunodeficiency virus (HIV) testing, and lumbar puncture. Further cerebrospinal fluid (CSF) analyses for Venereal Disease Research Laboratory (VDRL) and cryptococcal antigen also returned negative. Consequently, the decision was made to transfer the patient to a hospital with inpatient rheumatology for further evaluation of encephalopathy versus acute psychosis in the setting of SLE. 

**Figure 1:**
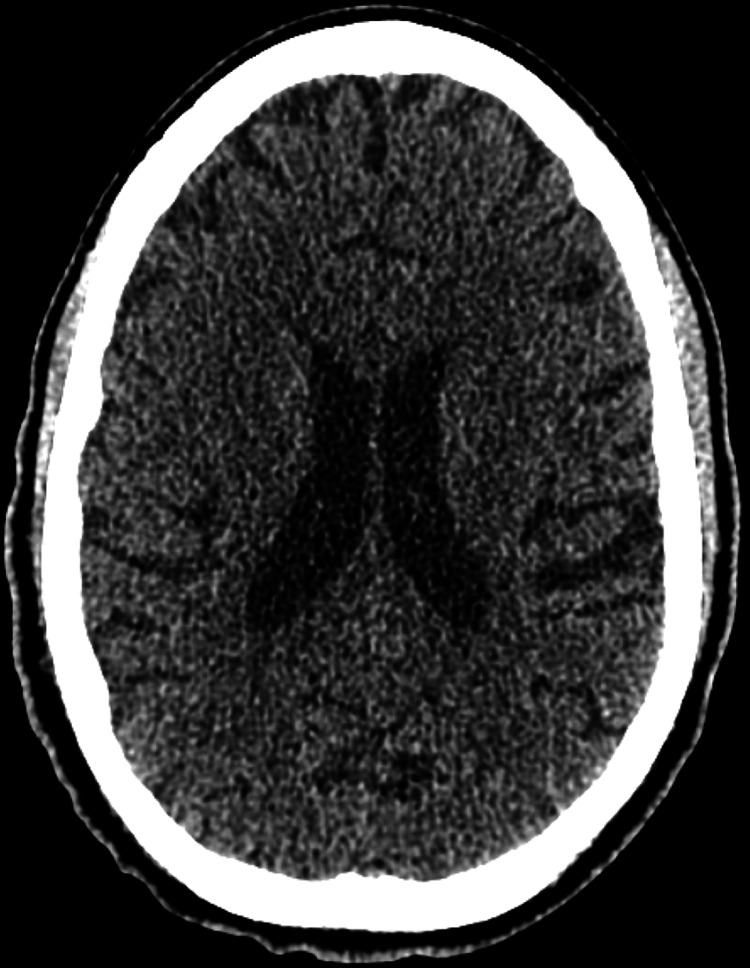
CT scan without intravenous contrast of the head showing no hemorrhage or acute large territorial infarct. The ventricles and basal cisterns are patent. There is no hydrocephalus or midline shift. Overall, no acute intracranial abnormality.

Upon arrival, vital signs indicated a temperature of 102.4 degrees Fahrenheit, a heart rate of 125 beats per minute, a respiratory rate of 20 breaths per minute, and an oxygen saturation of 97% on room air. During the physical examination, the patient appeared distressed and exhibited involuntary movements of her extremities. She did not display any focal neurologic deficits but was unresponsive verbally, only offering intermittent nods in response to questions. Additionally, she presented with bilateral rotary nystagmus and was unable to follow finger tracking. The remainder of the examination yielded normal findings. CT scan of the head was also noncontributory (Figure 1).

The results of initial laboratory testing are revealed in Table 1. Notably, her ANC was dramatically reduced to 340 (reference range: 1.5-8.0 x 10^9^ cells/L), alongside severe pancytopenia. Urinalysis suggested significant proteinuria with a urine protein/creatinine ratio of 5.94 mg/g (reference range: ≤ 200 mg/g). The iron panel displayed a significantly elevated ferritin level of 3,520 ng/mL (reference range: 15.0-150 ng/mL). Additionally, hypertriglyceridemia of 328 mg/dL (reference range: ≤ 150 md/dL) was noted. Coagulation studies revealed fibrinogen 214 mg/dL (reference range: 200-400 mg/dL), lactate dehydrogenase 919 IU/L (reference range: 126-266 IU/L), and an undetectable haptoglobin with normal prothrombin time (PT) and international normalized ratio (INR). Autoimmune studies indicated decreased but improving complement component 3 (C3) at 55 mg/dL (reference range: 82-167 mg/dL) and an improving anti-double-stranded deoxyribonucleic acid antibody (anti-dsDNA) level from her prior at 75 IU/mL (reference range: ≥ 10 IU/mL, positive). Complement component 4 (C4) in addition to repeated anti-cardiolipin antibody and beta-2-glycoprotein studies were within normal limits.

**Table 1:** Initial laboratory testing upon presentation along with autoimmune workup

Complete metabolic panel	Value	Reference Range
Sodium	142	135 - 145 mmol/L
Potassium	4.3	3.3 - 4.6 mmol/L
Chloride	112	101 - 110 mmol/L
Carbon Dioxide	20	21 - 29 mmol/L
Urea Nitrogen	19	6 - 22 mg/dL
Creatinine	0.89	0.51 - 0.96 mg/dL
Blood Urea Nitrogen (BUN)/Creatinine Ratio	21.3	6 - 22
Glucose	105	71 - 99 mg/dL
Calcium	8.1	8.6 - 10.0 md/dL
Total Protein	5.3	6.5 - 8.3 g/dL
Albumin	2.4	3.8 - 4.9 g/dL
Total Bilirubin	0.2	0.2 - 1.0 mg/dL
Alkaline Phosphatase	38	35 - 104 IU/L
AST	108	14 - 33 IU/L
ALT	35	10 - 42 IU/L
Anion Gap	10	4 - 16 mmol/L
EGFR	96	≥ 60 mL/min/1.73M2
Complete Blood Count and Differential		
WBC	1.19	4.0 - 10.0 x10^3^/µL
RBC	2.87	4.0 - 5/2 x10^6^/µL
Hemoglobin	7.5	12.0 - 16.0 g/dL
Hematocrit	23.6	35.0 - 45.0%
MCV	82.2	78.0 - 100.0 fL
MCH	26.1	26.0 - 34.0 pg
MCHC	31.8	31.0 - 36.0 g/dL
RDW	13.6	11.0 - 14.6%
Platelet Count	59	150 - 450x10^3^/µL
MPV	10.9	9.5 - 12.2 fL
Neutrophil %	23	34 - 73%
Bands %	5.3	0 - 10%
Lymphs %	70	25 - 45%
Monocytes %	1	2 - 6%
Metamyelocytes %	1	≤ 0%
Myelocytes %	0	≤ 0%
Promyelocytes %	0	≤ 0%
Atypical Lymphocytes %	0.0	0 - 10%
Neutrophil Absolute	0.34	1.4 - 7.5x10^3^/µL
Lymphocyte Absolute	0.83	0.7 - 3.1x10^3^/µL
Monocytes Absolute	0.01	0.1 - 0.9x10^3^/µL
Immature Granulocytes Absolute	0.01	≤ 0.0x10^3^/µL
Autoimmune		
C3	55	82 - 167 mg/dL
C4 serum compliment	19	10 - 40 mg/dL
Anti-double stranded deoxyribonucleic acid antibody (anti-dsDNA)	75	≥ 10 IU/mL, positive
Anti-cardiolipin antibody (IgG)	<9	≤ 10.0 GPL U/mL, negative
Anti-cardiolipin antibody (IgM)	10	≤ 13.0 MPL U/mL, negative
Beta-2-Glycoprotein IgG	<9	≤ 20 GPI IgG units
Beta-2-Glycoprotein IgM	<9	≤ 32 GPI IgM units

**Figure 2:**
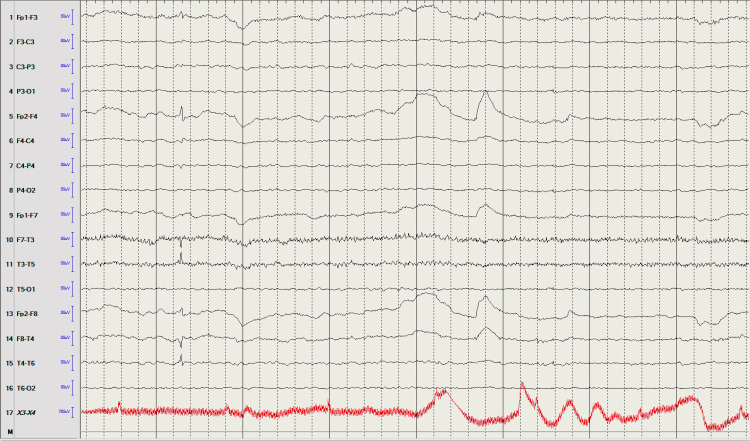
EEG interpretation When the patient was awake, there was a background of 10-11 hertz alpha, which was noted posteriorly and symmetric bilaterally with attenuation to eye-opening. Significant movement and muscle artifacts were noted intermittently during the record. As the recording progressed, the patient became drowsy with dropout of the background and the appearance of more diffuse beta. No definite epileptiform discharges or electrographic seizures were noted during the recording. Photic stimulation was not performed. This is a normal awake and drowsy EEG.

**Figure 3:**
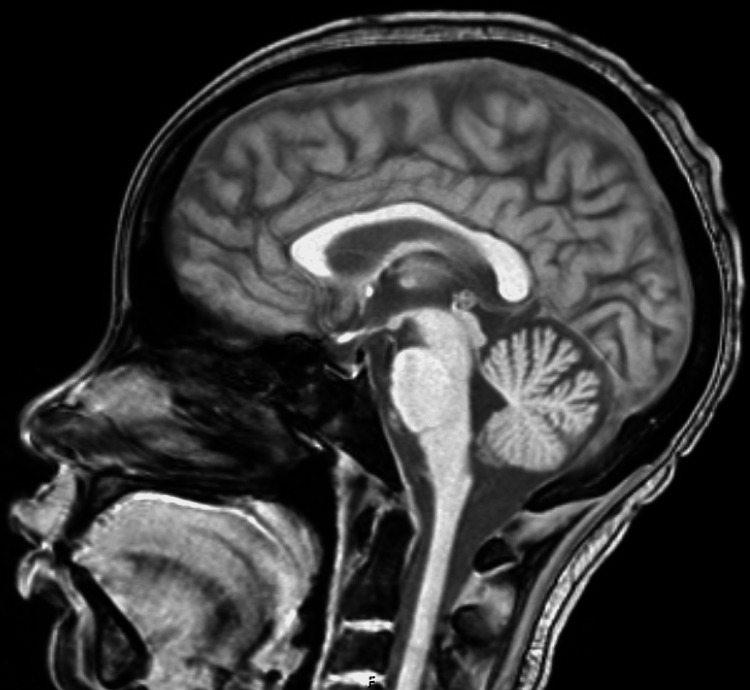
MRI of the brain with and without contrast with a T1-weighted image in the sagittal view showing a negative exam for acute infarct, intracranial hemorrhage, extra-axial collection, midline shift, or hydrocephalus. There is no abnormal enhancement or evidence of tumor. Major intracranial flow voids are present. There is normal enhancement of the major dural venous sinuses on high-resolution imaging. Overall, no acute intracranial abnormality.

Chest x-ray was concerning for possible right middle and lower lobe pneumonia. The patient was placed on neutropenic precautions and broad-spectrum antibiotics were continued. Continuous EEG did not demonstrate any seizure or epileptiform activity (Figure 2). MRI brain with and without contrast also demonstrated no acute abnormalities (Figure 3). Lumbar puncture was unrevealing with the results of CSF studies displayed in Table 2.

**Table 2:** Lumbar puncture CSF studies that were noncontributory

CSF studies	Value	Reference
Appearance, Fluid	Hazy	Clear, colorless
Fluid Color	Red	Clear, colorless
RBC Count, Fluid	6,733	≤ 0/µL
WBC Count, Fluid	2	0-10/µL
Glucose	45	6-80 mg/dL
Protein	27	15-45 mg/dL
HSV NAA	Not Detected	Not Detected
VZV NAA	Not Detected	Not Detected

Given the findings of her fever, pancytopenia, and significantly elevated ferritin with hypertriglyceridemia, concern for MAS was raised, and 1 g of intravenous (IV) methylprednisolone daily was initiated. Due to a promising clinical response to this regimen, diagnosis of MAS was felt to be more likely, and the decision was made to start anakinra.

Surveillance CT chest, abdomen, and pelvis did not show any signs of pneumonia or intra-abdominal infection but demonstrated bilateral pleural effusions and anasarca, consistent with capillary leak secondary to MAS. Remaining infectious evaluation including respiratory viral pathogen panel, repeat blood cultures, urine culture, tuberculin skin testing, Epstein Barr virus viral capsid antigen immunoglobulin (Ig) M, coxsackie IgM, and IgG testing, urine Histoplasma galactomannan antigen, serum aspergillus antigen, and serum human polyomavirus 2 were negative as noted in Table 3.

**Table 3:** Unremarkable infectious workup investigation

Infectious workup	Value	Reference
Blood Cultures	Negative	Negative
Respiratory viral pathogen panel	Not Detected	Not Detected
S pneumoniae Urine Antigen	Negative	Negative
Legionella Urine Antigen	Negative	Negative
Histoplasma Urine Antigen	Negative	Negative
Trichomonas Vaginalis	Negative	Negative
Yeast, Urine	Negative	Negative
Urine Culture	Negative	Negative
HIV ½ Antigen/Antibody Screen	Negative	Negative
Tuberculin skin testing	Negative	Negative if 0 mm induration
Epstein-Barr Virus viral capsid antigen immunoglobulin IgM	≤ 36	0.0 - 35.9 U/mL
Coxsackie immunoglobulin A7, A9, A16, A24 IgG	Negative	Negative < 1:100 titer
Coxsackie immunoglobulin A7, A9, A16, A24 IgM	Negative	Negative < 1:10 titer
Serum Human Polyomavirus 2	Negative	Negative
Aspergillus Galactomannan, Blood	0.07	0.00 - 0.49 Index

A bone marrow biopsy revealed a hypocellular marrow with multilineage hematopoiesis and maturation but no overt evidence of involvement by acute leukemia, lymphoma, high-grade myelodysplasia, or hemophagocytic histiocytes in the core biopsy. Subsequently, the interleukin-2 (IL-2) receptor alpha level returned elevated at 757 U/mL. 

After ruling out infectious and paraneoplastic causes, MAS remained the primary diagnosis. The patient finished her course of antibiotics and remained without fever. Treatment with a prednisone taper and anakinra led to significant clinical improvement, returning the patient to her baseline mental status and resolving inflammatory markers, liver function abnormalities, and pancytopenia.

## Discussion

MAS, also referred to as secondary HLH, represents a rare and life-threatening complication of rheumatologic disease poorly studied in the adult population [2]. While commonly associated with AOSD and sJIA, MAS can manifest in other rheumatologic conditions such as Kawasaki disease and SLE [1,3-6]. Though approximately 10% of children with sJIA progress to overt MAS, and up to 40% exhibit a milder, often subclinical form, MAS incidence is notably lower in adults [7]. According to Gilboa et al.'s decade-long database investigation, the median age of MAS onset is 32, with more than half of patients concurrently presenting with an underlying rheumatologic disease [8]. In the case of our 18-year-old patient, she was diagnosed with lupus at age 15 with ANA 1:1,280 titer, anti-smith antibody > 8 (reference range < 1.0 negative AI), and double-stranded DNA antibody 172 (reference range >10 positive). 

MAS is characterized by a dysfunctional immune response that leads to cytotoxic dysfunction and uncontrolled activation and proliferation of T lymphocytes and macrophages. The activated immune cells produce large amounts of proinflammatory cytokines, including interleukin (IL)‐1β and interleukin 6 (IL‐6), creating a cytokine storm that can lead to multi-organ dysfunction and failure [8-10]. 

The clinical presentation of HLH and MAS encompasses a wide spectrum of symptoms with low specificity, necessitating a heightened level of suspicion [10]. Distinguishing HLH from conditions like sepsis, a frequently encountered life-threatening emergency, can be challenging due to shared features such as fever and hyperinflammation, alongside a generally unwell appearance in patients [11]. Machowicz et al. proposed a diagnostic approach aimed at distinguishing HLH in patients with suspected sepsis, focusing on key differentiating factors, notably ferritin, as the primary diagnostic marker and then evaluating for splenomegaly and cytopenias [5,11,12]. This approach was particularly noteworthy and beneficial considering HLH/MAS lacks a singular discriminating factor from sepsis [11]. This parallels the experience of our patient who underwent extensive investigations for infectious diseases and imaging to identify the source of neurological dysfunction.

This unusual MAS case posed diagnostic challenges due to its atypical neurologic presentation in the setting of known lupus. Neurologic dysfunction is a prevalent initial indication of HLH, with reported manifestations including irritability, altered mental status, seizures, cranial nerve palsies, hemiplegia, or encephalitis [10]. In our patient, the neurologic symptoms presented as hallucinations, paranoid delusions, insomnia, and erratic behavior, manifestations less frequently documented in the literature. The patient’s long-term steroid therapy and altered mental status with hallucinations questioned the diagnosis of steroid-induced psychosis or lupus cerebritis. Steroid-induced psychosis, however, is seen with oral steroids and more commonly witnessed with higher doses, compared to our patient on prednisone 50 milligrams daily [13]. Cessation or reduction of steroids usually leads to the resolution of psychotic symptoms, which was not the case in this patient [14]. Lupus cerebritis, moreover, is a diagnosis of exclusion. Given the improvement in lupus markers, absence of structural brain abnormalities, negative EEG findings, lack of history of drug use, and no identifiable source of infection, it was unlikely to precipitate our patient’s complex presentation.

The diagnostic criteria are determined by the HLH-2004 trial, which requires the presence of at least five of the eight following features: fever ≥38.5°C, splenomegaly, peripheral blood cytopenia, hypertriglyceridemia and/or hypofibrinogenemia, hemophagocytosis in bone marrow, spleen, lymph node, or liver, low or absent natural killer (NK) cell activity, ferritin >500 ng/mL, elevated soluble CD25 (soluble IL-2 receptor alpha) two standard deviations above age-adjusted laboratory-specific norms [10]. To enhance the recognition of MAS in juvenile SLE patients, a multinational multicenter study conducted a comparison between patients exhibiting macrophage hemophagocytosis on bone marrow biopsy with those who did not. The study determined that hyperferritinemia provided the highest sensitivity and specificity, followed by lactate dehydrogenase, hypertriglyceridemia, and hypofibrinogenemia [5]. Another study identified a statistically significant correlation between elevated levels of interleukin-2 receptor alpha (sIL-2Ralpha) and soluble CD163 (sCD163) in MAS patients compared to those with sJIA [15]. Among hyperferritinemia adults, other potential differentials include septic shock, liver disease, and hematologic malignancies; however, the HLH-2004 criteria should ultimately be applied to exclude HLH [12].

The management of MAS necessitates a multidisciplinary team approach consisting of hematology-oncology with rheumatology. Treatment entails addressing laboratory abnormalities through blood or platelet transfusions as needed, along with systemic glucocorticoids and anakinra in select cases [16]. In refractory cases, cyclophosphamide has demonstrated clinical efficacy in mitigating the immune response, which was not an option for our end-stage renal disease patient [17]. Despite advancements, the pathogenesis underlying elevated levels of sCD163 from macrophages and heightened IL-6, IL-18, ferritin, and interferon-gamma from NK cells remains poorly understood [2]. The favorable response observed with anakinra, an IL-1 inhibitor, underscores the need for further exploration to optimize treatment outcomes [18].

The survival of patients with HLH/MAS depends on prompt diagnosis and treatment. Even if test results are pending, or not all five of the eight necessary diagnostic criteria are met, treatment should not be postponed if HLH is high on the differential diagnosis. Because there are no controlled trials on MAS treatment available, the management of this syndrome is primarily empirical and based on short case series, the majority of which include children [16,18,19]. Despite treatment, the persistent hyperinflammatory state precipitating a cytokine storm can lead to multiorgan failure, thus imparting a substantial mortality risk in MAS cases [2,8,20]. Research by Lambotte et al. indicates that SLE-associated hemophagocytic syndrome (HS) may delineate a more severe variant of SLE, heightening the risk of flares and recurrence [17]. While further investigations are warranted concerning prognostic indicators and therapeutic approaches, the presence of unexplained fever and cytopenia accompanied by hyperferritinemia in juvenile SLE patients should inherently trigger suspicion for MAS [5]. Data are scarce on the prognosis, management, and clinical features of MAS in adults. 

## Conclusions

MAS represents a rare rheumatological complication with potential fatality. Its differential diagnosis spans a wide range, demanding a heightened clinical suspicion, especially in high-risk patients. Elevated ferritin levels have emerged as the most consistent and reliable laboratory finding. Confirmation entails bone marrow biopsy, with treatment typically involving steroids and anakinra. The unique presentation of our patient posed diagnostic challenges, highlighting the varied presentations of this condition and emphasizing the necessity for further research on this uncommon disorder.
